# Antibacterial Activity of Blue Light against Nosocomial Wound Pathogens Growing Planktonically and as Mature Biofilms

**DOI:** 10.1128/AEM.00756-16

**Published:** 2016-06-13

**Authors:** Fenella D. Halstead, Joanne E. Thwaite, Rebecca Burt, Thomas R. Laws, Marina Raguse, Ralf Moeller, Mark A. Webber, Beryl A. Oppenheim

**Affiliations:** a Clinical Microbiology, Queen Elizabeth Hospital, University Hospitals Birmingham NHS Foundation Trust, Birmingham, United Kingdom; b NIHR Surgical Reconstruction and Microbiology Research Centre, Queen Elizabeth Hospital, Birmingham, United Kingdom; c Institute of Microbiology and Infection, University of Birmingham, Birmingham, United Kingdom; d Chemical, Biological and Radiological Division, Dstl, Porton Down, Salisbury, Wiltshire, United Kingdom; e German Aerospace Center (DLR e.V.), Institute of Aerospace Medicine, Radiation Biology Department, Space Microbiology Research Group, Cologne, Germany; Washington State University

## Abstract

The blue wavelengths within the visible light spectrum are intrinisically antimicrobial and can photodynamically inactivate the cells of a wide spectrum of bacteria (Gram positive and negative) and fungi. Furthermore, blue light is equally effective against both drug-sensitive and -resistant members of target species and is less detrimental to mammalian cells than is UV radiation. Blue light is currently used for treating acnes vulgaris and Helicobacter pylori infections; the utility for decontamination and treatment of wound infections is in its infancy. Furthermore, limited studies have been performed on bacterial biofilms, the key growth mode of bacteria involved in clinical infections. Here we report the findings of a multicenter *in vitro* study performed to assess the antimicrobial activity of 400-nm blue light against bacteria in both planktonic and biofilm growth modes. Blue light was tested against a panel of 34 bacterial isolates (clinical and type strains) comprising Acinetobacter baumannii, Enterobacter cloacae, Stenotrophomonas maltophilia, Pseudomonas aeruginosa, Escherichia coli, Staphylococcus aureus, Enterococcus faecium, Klebsiella pneumoniae, and Elizabethkingia meningoseptica. All planktonic-phase bacteria were susceptible to blue light treatment, with the majority (71%) demonstrating a ≥5-log_10_ decrease in viability after 15 to 30 min of exposure (54 J/cm^2^ to 108 J/cm^2^). Bacterial biofilms were also highly susceptible to blue light, with significant reduction in seeding observed for all isolates at all levels of exposure. These results warrant further investigation of blue light as a novel decontamination strategy for the nosocomial environment, as well as additional wider decontamination applications.

**IMPORTANCE** Blue light shows great promise as a novel decontamination strategy for the nosocomial environment, as well as additional wider decontamination applications (e.g., wound closure during surgery). This warrants further investigation.

## INTRODUCTION

Antimicrobial resistance (AMR) is rapidly evolving and emerging to be a large threat to modern medicine. Although affecting only a minority of admissions, health care-associated infections are associated with increased mortality, prolonged hospital stays, and increased treatment costs (1). With the rise in resistance to the carbapenem class of antibiotics in Gram-negative organisms (2), there is a significant threat of infections becoming wholly untreatable with current treatment regimens (3, 4).

Much research is now focused on alternatives to the conventional antimicrobial agents. These mostly involve topical agents (with the aim to reduce surface contamination and therefore lower the risks of sepsis and infection progression) with research to date on a large number of agents. Since the environment is a key source of nosocomial pathogens (5), there has also been renewed focus on hospital cleaning and disinfection, especially via antimicrobial chemicals delivered in a novel way, including antimicrobial light sources (1, 6). These novel strategies, capable of decontaminating both the patient's wound and the environment, have the potential to be highly beneficial in the fight against AMR and nosocomial infections.

The blue wavelengths within the visible light spectrum (especially wavelengths between 400 and 470 nm) are intrinsically antimicrobial and do not require additional exogenous photosensitizers to exert an antimicrobial effect (4). Photodynamic inactivation of both bacterial and fungal cells occurs as a result of photoexcitation of intracellular porphyrins (1) by blue light, leading to energy transfer and the production of highly cytotoxic reactive oxygen species (ROS), primarily singlet oxygen (^1^O_2_) (4, 7–9). All wavelengths from 400 to 425 nm can be used for microbial inactivation; however, the optimal antimicrobial activity occurs at 405 nm, since this is the point in the electromagnetic spectrum where maximum porphyrin excitation occurs (10). Although blue light is less germicidal than UV light (1), pathogens can be selectively inactivated without damaging human cells, and consequently, blue light is considered much less detrimental to mammalian cells than UV light (11, 12). One potential benefit of light-based antimicrobial therapies is equal efficacies against drug-sensitive and resistant members of target species (13, 14).

Blue light has been shown to exhibit a broad spectrum of antimicrobial effect against bacteria and fungi, although generally the Gram-positive bacteria are considered more susceptible to blue light than the Gram-negative bacteria (15, 16). Successful inactivation has been demonstrated *in vitro* against Staphylococcus aureus (including methicillin-resistant S. aureus [MRSA]), Clostridium difficile (both spores and vegetative cells), Acinetobacter baumannii, Escherichia coli, Staphylococcus epidermidis, Pseudomonas aeruginosa, Klebsiella pneumoniae, Streptococcus pyogenes, and Mycobacterium spp. (14, 15, 17, 18). In addition to the key nosocomial pathogens, blue light is also effective against Propionibacterium acnes and has been used topically to treat acne vulgaris (19, 20) and Helicobacter pylori, in which case blue light is used internally as a “light string” to treat stomach infections (21). Owing to the mechanism of action of blue light, it is unlikely that viruses will be susceptible unless photosensitizers are added to enhance virucidal activity (22).

The use of blue light for treatment of wound infections *in vivo* is an emerging technology. To date, blue light therapy has been shown to significantly reduce the bacterial burden of wounds infected with P. aeruginosa (23), MRSA (24), and A. baumannii (25), and it saved the lives of mice subjected to potentially lethal burns contaminated with P. aeruginosa and A. baumannii (23, 25).

As well as having clinical application for patient treatment, blue light is also a promising candidate for the control of problematic microorganisms in the clinical setting (e.g., the disinfection of air and exposed surfaces). In this regard, Bache et al. (26) and Maclean et al. (1) have performed studies with a new disinfection technology termed the HINS-light environmental decontamination system (EDS) which delivers low-irradiance 405-nm light continuously and is suitable for use in patient-occupied settings. Evaluation studies performed by the latter authors showed that there was a statistically significant 90% reduction in numbers of culturable Staphylococci spp. following 24 h of use in an unoccupied room (5) and reductions of 56 to 86% when used in burn isolation rooms occupied by MRSA-positive patients. Furthermore, when the system was no longer used, the room became recontaminated to levels similar to those pretreatment.

The vast majority of research on blue light has been carried out on bacteria in the planktonic phase, dispersed evenly in a liquid medium. In nature this is rarely the case, since most bacteria aggregate to form complex communities within a matrix of extracellular polymeric substances termed a biofilm. There are many advantages for this compared to planktonic growth, including increased resistance to killing via antimicrobials, immune cells, chemicals, and environmental stresses (27). Furthermore, once a biofilm has become established on a surface, it is extremely hard to eradicate. Medically, biofilms have been associated with a myriad of chronic infections, acute infections, colonization of indwelling medical devices, and wound infections (27–29).

Since we know that the majority of clinical infections and environmental contamination involve microbial biofilms (30), this multicenter *in vitro* study was performed to assess the antibacterial activity of blue light against biofilms of a range of important nosocomial pathogens.

## MATERIALS AND METHODS

A series of *in vitro* experiments were conducted with a panel of organisms (Table 1) to determine the efficacy of blue light (400 nm) against bacteria in planktonic (free-floating in broth) and biofilm (attached to a surface) modes of growth. The panel comprised well-characterized control and clinical isolates (in terms of their antibiograms and abilities to form biofilms *in vitro*) and concentrated mostly on A. baumannii strains from a protracted outbreak at the Queen Elizabeth Hospital in Birmingham (QEHB) (31). A. baumannii is a key nosocomial pathogen which survives in hospital and health care environments despite conditions such as desiccation, nutrient starvation, and antimicrobial chemicals (e.g., disinfectants) (32, 33). Despite stringent infection control practices, a large outbreak of A. baumannii occurred at QEHB, where 65 patients tested positive during the outbreak period (July 2011 to February 2013). The strains from this outbreak demonstrated a high degree of resilience in the hospital environment, and there was also evolution among the isolates over time to increase desiccation resistance and biofilm formation capacity. Additional A. baumannii isolates (representing genetically diverse strains) were tested in this panel to add some diversity to the strains, including strains ACI_AYE (a representative of international clone I, a major globally relevant lineage), ACI_C60 (a control strain of a unique pulsed-field gel electrophoresis [PFGE] type), and ACI_19606 (a control strain of a further unique PFGE type) (typing data not shown).

**TABLE 1:** Clinical and control isolates used in this study^*a*^

Study identifier	Organism	Description
ACI_616	Acinetobacter baumannii	QEHB clinical outbreak isolate
ACI_618	Acinetobacter baumannii	QEHB clinical outbreak isolate
ACI_642	Acinetobacter baumannii	QEHB clinical outbreak isolate
ACI_648	Acinetobacter baumannii	QEHB clinical outbreak isolate
ACI_659	Acinetobacter baumannii	QEHB clinical outbreak isolate
ACI_665	Acinetobacter baumannii	QEHB clinical outbreak isolate
ACI_671	Acinetobacter baumannii	QEHB clinical outbreak isolate
ACI_672	Acinetobacter baumannii	QEHB clinical outbreak isolate
ACI_698	Acinetobacter baumannii	QEHB clinical outbreak isolate
ACI_AYE	Acinetobacter baumannii	MPR clinical isolate (unique)
ACI_C60	Acinetobacter baumannii	NCTC 13424 (unique)
ACI_19606	Acinetobacter baumannii	ATCC 19606 (unique)
ENTCL_525	Enterobacter cloacae complex	QEHB clinical isolate
ENTCL_801	Enterobacter cloacae complex	QEHB clinical isolate
ENTCL_804	Enterobacter cloacae complex	QEHB clinical isolate
STEMA_529	Stenotrophomonas maltophilia	QEHB clinical isolate
STEMA_551	Stenotrophomonas maltophilia	QEHB clinical isolate
STEMA_558	Stenotrophomonas maltophilia	QEHB clinical isolate
PSE_568	Pseudomonas aeruginosa	QEHB clinical isolate
PSE_PA01	Pseudomonas aeruginosa	ATCC 15692
PSE_6749	Pseudomonas aeruginosa	NCTC 6749
PSE_1054	Pseudomonas aeruginosa	QEHB clinical burn isolate
PSE_1586	Pseudomonas aeruginosa	QEHB clinical burn isolate
EKIN_502	Elizabethkingia meningoseptica	QEHB clinical isolate
EC_073	Escherichia coli	EPEC CFT_073
EC_042	Escherichia coli	EAEC_042
MDR_A	CPE Klebsiella pneumoniae (NDM-1 positive)	QEHB clinical isolate
MDR_B	CRE Klebsiella pneumoniae (ESBL positive with additional permeability changes)	QEHB clinical isolate
MRSA_508	Staphylococcus aureus	QEHB clinical isolate
MRSA_520	Staphylococcus aureus	QEHB clinical isolate
MRSA_531	Staphylococcus aureus	QEHB clinical isolate
MSSA_10788	Staphylococcus aureus	NCTC 10788
MSSA_F77	Staphylococcus aureus	NCTC 8532
EFM_513	Enterococcus faecium	QEHB clinical isolate
MSSA_29213	Staphylococcus aureus	ATCC 29213
MSSA_10442	Staphylococcus aureus	NCTC 10442
MSSA_33807	Staphylococcus aureus	ATCC 33807
MSSA_4163	Staphylococcus aureus	NCTC 4163

a MPR, Ministry of Research, Paris; CPE, carbapenemase-producing Enterobacteriaceae; NDM-1, New Delhi metallo-β-lactamase; CRE, carbapenem-resistant Enterobacteriaceae; ESBL, extended-spectrum β-lactamase.

We also tested a small range of other comparator organisms commonly causing hospital-acquired infections, including Enterobacter cloacae, Stenotrophomonas maltophilia, P. aeruginosa, E. coli, S. aureus, and Enterococcus faecium, and we included control strains (PS_6749 and MSSA_10788) recognized in the EN standards for assessing the efficacy of chemical disinfectants (e.g., EN 13727 [34]). The panel comprised isolates that in previous tests had demonstrated ability to form relevant quantities of biofilm *in vitro* and furthermore included two carbapenem (multidrug)-resistant isolates of K. pneumoniae and a single isolate of Elizabethkingia meningoseptica from a wound swab. This is an intrinsically highly resistant organism, usually resistant to extended-spectrum β-lactam agents (due to production by most strains of two β-lactamases: one extended-spectrum β-lactamase [ESBL] and one class B carbapenem-hydrolyzing metallolactamase), aminogylcosides, tetracycline, and chloramphenicol (35).

All isolates were stored at −80°C on Protect beads and were routinely cultured on cysteine lactose electrolyte-deficient (CLED) agar or blood agar prior to each experiment.

Experiments were designed to assess the antibacterial activity of blue light against planktonic and biofilm growth forms of the panel of bacteria described above. Testing was performed at the Defense Science Technology Laboratory (Dstl) (planktonic growth) and the Surgical Reconstruction and Microbiology Research Centre (biofilms), and blue light of a 400-nm wavelength was used for all experiments.

### Blue light equipment.

High-intensity blue light was provided by a LED flood array (Henkel-Loctite, Hemel Hempstead, United Kingdom). This array utilizes 144 reflectorized LEDs which produce a homogeneous illuminated area of 10 cm by 10 cm. The emission spectrum of the LED array was determined using a USB2000 spectrophotometer (Ocean Optics, Oxford, United Kingdom). Two identical platforms were used for the testing, both of which were calibrated at Dstl using a PM100D radiant power meter (Thorlabs, Newton, NJ) prior to *in vitro* testing to ensure a reproducible irradiance of 60 mW/cm^2^ when the LED array is positioned 15.5 cm above the test area. All of the experimental conditions (except wavelength) adhere to the optimal criteria outlined by Coohill and Sagripanti (36) for the assessment of bacterial sensitivity to UV-C radiation.

### Impact of blue light on planktonic bacteria.

Bacterial isolates were grown overnight in Luria broth (LB; Sigma-Aldrich, United Kingdom) and then diluted in sterile phosphate-buffered saline (PBS) to produce a starting concentration of approximately 1 × 10^6^ bacteria per ml. Test samples (2 ml) were inoculated into a 12-well microtiter plate (Corning, NY), sealed with an optically clear ABsolute quantitative PCR (qPCR) sealer (Thermo Fisher Scientific, Paisley, Scotland) to prevent evaporation, and then exposed to blue light for 30 min (samples were taken for viable cell counting at 5-min intervals). If the strains still showed viability after 30 min of blue light exposure, the test was repeated over 180 min, with samples taken at 20-min intervals. An identical dark control plate was set up, wrapped in aluminum foil, and placed in the flood array adjacent to the blue light-irradiated samples.

At time increments during the experiment, samples exposed to blue light and incubated in the dark were removed and viable bacteria enumerated by serial dilution and growth on LB agar plates. The blue light sensitivity for each strain was determined from the mean of three independent biological replicates, with two technical replicates within each experiment.

The blue light dose (joules per square centimeter) received by the bacteria was calculated by multiplying the irradiance of light (watts per square centimeter) to which the sample was exposed by the exposure time (seconds).

### Impact of blue light on preformed biofilms.

The antibacterial activity of blue light against preformed biofilms was assessed by conducting minimum biofilm eradication concentration (MBEC) experiments (37) on each isolate. Overnight LB cultures of the test strains (made by inoculating approximately three to five colonies into 5 ml of fresh LB and incubating them at 37°C overnight) were diluted in fresh antibiotic-free Mueller-Hinton (MH) broth to an optical density at 600 nm (OD_600_) of 0.1, and then 200 μl was seeded into wells of a 96-well microtiter tray (MTT). Positive (200 μl of organisms diluted to an OD_600_ of 0.1) and negative (200 μl of MH broth) controls were included per blue light time point to be tested.

To produce a “transferable biofilm,” a 96-well polypropylene plate (Starlabs, United Kingdom) was then placed into the MTT so that each well contained a “peg” on which biofilms could form, before the plates were sealed, and statically incubated at 33°C for 72 h. After 72 h, the pegs (±biofilm) were removed and washed in an MTT containing sterile water (to remove any unbound cells). The negative control (sterile broth only) “peg plate” was then placed in a clean, empty MTT and wrapped in foil. Following this, both the negative control and the positive test peg plate (bacteria only) were placed in the test area (15.5.cm beneath the light source) and exposed to the blue light for periods of 15, 30, 45, or 60 min (corresponding to a blue light dose of 54, 108, 162, and 216 J/cm^2^, respectively). The foil around the control plate prevented the pegs from receiving any blue light treatment (and hence these positive-control biofilms were not exposed to the blue light), but the control plate biofilms would have most likely been exposed to the same amount of heating and drying as the blue light-exposed test plate.

After the treatment, the peg plates were carefully placed into an MTT containing 200 μl of sterile MH broth (herein referred to as reporter broth) for overnight incubation. After 18 h, the OD of the reporter broth was measured to assess the viability (seeding) of the biofilms following blue light exposure.

To demonstrate the presence of biofilms on the pegs, crystal violet (CV) assays were additionally performed on the pegs after the OD of the reporter broth had been measured. This involved placing the pegs into MTTs containing 200 μl of 1% CV (which binds to any present microbial biomass of biofilm), followed by washing (to remove unbound CV) and solubilization of the CV in 200 μl of 70% ethanol. The peg biofilm biomass could then be measured using OD readings as done previously and the presence of the biofilm confirmed. Two biological and 10 technical replicates were performed for each strain and blue light exposure duration, respectively.

### Statistical analysis. (i) Planktonic tests.

For the planktonic data, the surviving fraction was determined from the quotient *N*/*N*_0_, with *N* being the number of colony formers of the irradiated sample and *N*_0_ that of the nonirradiated controls. Plotting the logarithm of *N*/*N*_0_ as a function of dose (blue light fluence in joules per square centimeter) allowed survival curves to be obtained.

To determine the curve parameters, the following relationship was used: ln *N*/*N*_0_ = IC × *F* + *n*, where *N* is the number of colony formers after blue light irradiation, *N*_0_ is the number of colony formers without irradiation, IC is the inactivation constant (in joules per square centimeter), *F* is fluence, and *n* is the extrapolation number (i.e., the intercept with the ordinate of the extrapolated semilog straight line). The inactivation constant and the reciprocal lethal dose (LD) values were determined from the slope of the dose-effect curves (linear portion of the curve).

To allow for comparison with other bactericidal radiation sources in the literature, blue light-mediated killing was calculated in terms of the inactivation constant slope (IC) and the kill kinetics, shown as the doses required to kill 37% and 90% of the bacterial cells (LD_37_ and LD_90_, respectively). The significance of the difference of the dose-effect curves was statistically analyzed using Student's *t* test. Differences with *P* values of <0.05 were considered statistically significant.

LD_90_ values were analyzed using the statistical software package IBM SPSS V21.0, were found to be log normal by QQ plot (data not shown), and were consequently transformed to the logarithm of 10 prior to parametric analysis. Differences between bacterial species was investigated using a 1-way analysis of variance (ANOVA), and the suitability of the data for parametric analysis was further established with the use of Levene's test for unequal variance (*P* = 0.165). Where only one bacterial strain of a species was available, this species was taken out of the analysis. Multiple comparisons were made using Bonferroni's correction. Similarly, the effect of pigmentation of S. aureus strains on susceptibility to blue light was tested by using *t* tests without Welch's correction, and suitability was further tested using Levene's test for unequal variance (*P* = 0.984).

### (ii) Biofilm tests.

The ability of biofilms to seed new growth following exposure to blue light was assessed by comparing the OD values at each blue light time point versus that of the untreated (positive) control, and significance was determined using Student's *t* test. In order to investigate any possible link between biofilm size and depth (colorimetry) and blue light sensitivity, these two parameters were investigated through QQ plots in SPSS (SPSS Statistics for Windows, version 21.0, IBM Corp., Armonk, NY). Initial analysis suggested that a transformation of both parameters by the logarithm of 10 was needed to render the data suitable for parametric analysis data not shown). Very little difference between the technical replicates was observed with regard to either parameter, and therefore, the median of the log_10_ of the technical replicates was used for analysis. The capacity for each strain to form a biofilm was taken from the average of the OD values over the 4 time points for the positive control. Comparisons of biofilm values were made using 1-way ANOVA and Student's *t* tests (without Welch's correction), and comparisons of variances were made with F tests and Brown-Forsythe tests. The viability of each strain of bacteria in biofilm was analyzed by Bonferroni's posttests across each time point (SPSS). Where significant differences between the positive control and the blue light occurred, the blue light was regarded as having an effect from there on, leading to an ordinal score for each strain of 15 min (54 J/cm^2^), 30 min (108 J/cm^2^), 45 min (162 J/cm^2^), 60 min (216 J/cm^2^), or >60 min. Comparisons of biofilm sensitivity scores were made using Kruskal-Wallis tests.

In order to characterize whether correlation existed between measured parameters, Spearman's method was used. In order to determine the statistical power of the correlations, the computer program SPSS sample power V3.0 (IBM) was used, and power was calculated for one sample correlations using the derived *R* value and the sample size (*n =* 34).

## RESULTS

Blue light was tested against 34 bacterial isolates, including clinical isolates from QEHB and culture collection type strains. The results of the spectral output testing of the blue light platform (with an Ocean Optics USB2000 spectrometer) determined that the emission peak of the blue light produced was at 400 nm, with a full-width half-maximum value of ±8.5 nm (see Fig. S1 in the supplemental material).

### Sensitivity of isolates to blue light when grown in planktonic culture.

**FIG 1:**
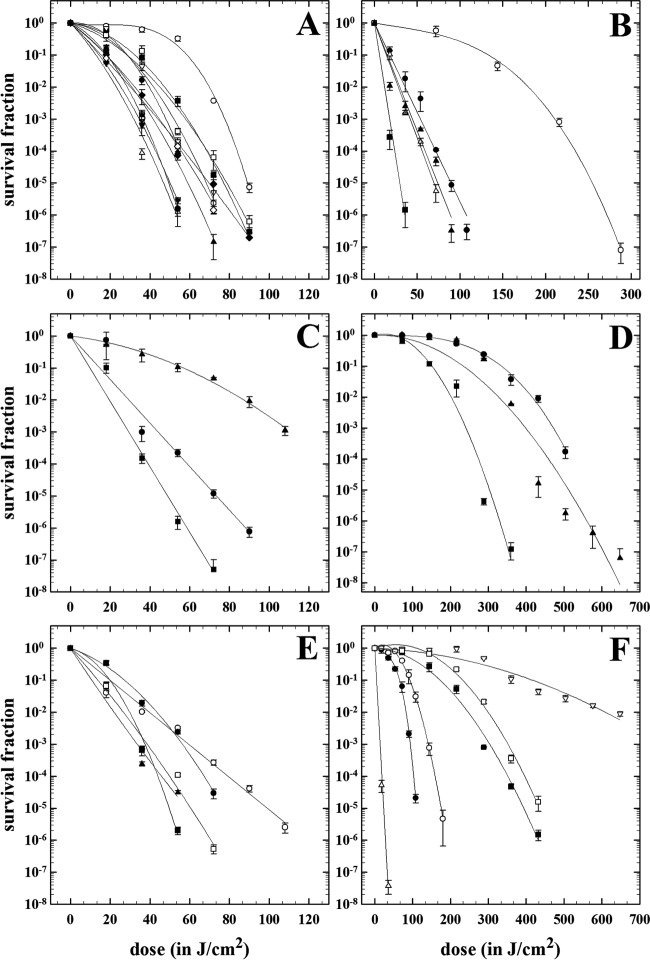
Survival of planktonic bacteria after exposure to 400-nm blue light. (A) Acinetobacter baumannii strains are represented as follows: ACI_616, closed circles; ACI_618, open circles; ACI_AYE, closed triangles; ACI_665, open triangles; ACI_19606, closed inverted triangles; ACI_648, open inverted triangles; ACI_659, closed diamonds; ACI_C60, open diamonds; ACI_671, closed squares; ACI_672, open squares; ACI_698, closed hexagons; and ACI_642, open hexagons. (Note that the hexagons are behind the other symbols.) (B) Staphylococcus aureus strains are represented as follows: MSSA _10788, open circles; MSSA_F77, closed triangles; MRSA_520, closed squares; MRSA_531, open triangles; and MRSA_508, closed circles. (C) Stenotrophomonas maltophilia strains are represented as follows: STEMA_ 558, triangles; STEMA_551, circles; and STEMA_529, squares. (D) Enterobacter cloacae strains are represented as follows: ENTCL_804, circles; ENTCL_801, triangles; and ENTCL_525, squares. (E) Pseudomonas aeruginosa strains are represented as follows: PSE_1586, closed circles; PSE_PAO1, open circles; PSE_568, closed triangles; PSE_1054, closed squares; and PSE_6479, open squares. (F) Other strains are represented as follows: E. coli EC_042, open circles; E. coli EC_073, closed circles; K. pneumoniae MDR-A, open squares; K. pneumoniae MDR-B, closed squares; Elizabethkingia meningoseptica EKIN_502, open triangles; and Enterococcus faecium EFM_513, open inverted triangles. Data are averages ± standard deviations (*n =* 3).

All 34 isolates were sensitive to blue light treatment, and there was no significant decay in the dark incubated controls. In contrast, rapid and substantive loss of viability was observed where all test bacteria were exposed to blue light (Fig. 1).

Twenty-four of the isolates (71%) demonstrated at least a 5-log_10_ decrease in viability following 15 min (54 J/cm^2^) to 30 min (108 J/cm^2^) of blue light exposure (Table 2), and for the majority of these isolates (A. baumannii [12/12], S. aureus [4/5], S. maltophilia [2/3], and E. meningoseptica [1/1]), there was a >6-log_10_ decrease in viability. Ten of the 34 isolates showed a <5-log_10_ decrease in viability. The isolates concerned included E. cloacae (ENTCL_525, ENTCL_801, and ENTCL_804), E. coli (EC_073 and EC_042), K. pneumoniae (MDR_A and MDR_B), S. aureus (MSSA_10788), S. maltophilia (STEMA_551), and E. faecium (EFM_513). Four of the 34 isolates (E. cloacae ENTCL_525, ENTCL_801, and ENTCL_804 and E. faecium EFM_513) took longer to kill than the majority of isolates, requiring extended periods up to 120 min (432 J/cm^2^) to obtain 2- to 3-log_10_ decrease in viability.

**TABLE 2:** Antimicrobial effects of blue light on planktonic cells

Isolate	Exposure time (min)	Irradiance (mW/cm^2^)	Dose (J/cm^2^)	Log_10_ reduction	*P* value	LD_37_ value^*a*^ (J/cm^2^)	LD_90_ value^*a*^ (J/cm^2^)
ACI_616	30	60	108	7.06	0.006	21 ± 2	27 ± 2
ACI_618	30	60	108	5.78	0.007	55 ± 4	59 ± 3
ACI_642	30	60	108	6.73	0.006	9 ± 1	16 ± 2
ACI_648	30	60	108	6.14	0.007	21 ± 2	29 ± 3
ACI_659	30	60	108	6.55	0.006	8 ± 1	16 ± 2
ACI_665	30	60	108	6.14	0.006	7 ± 1	12 ± 1
ACI_671	30	60	108	6.34	0.006	25 ± 2	32 ± 4
ACI_672	30	60	108	6.22	0.006	16 ± 2	24 ± 2
ACI_698	30	60	108	6.39	0.008	9 ± 1	14 ± 2
ACI_AYE	30	60	108	6.70	0.006	10 ± 1	16 ± 2
ACI_C60	30	60	108	6.76	0.007	7 ± 1	14 ± 1
ACI_19606	30	60	108	6.81	0.006	7 ± 1	13 ± 1
ENTCL_525	100	60	360	6.76	0.006	113 ± 12	136 ± 19
ENTCL_801	180	60	648	6.61	0.009	212 ± 20	246 ± 25
ENTCL_804	160	60	576	6.24	0.007	258 ± 18	306 ± 24
STEMA_529	30	60	108	7.21	0.006	7 ± 1	12 ± 2
STEMA_551	30	60	108	2.97	0.006	26 ± 3	48 ± 5
STEMA_558	30	60	108	7.33	0.006	8 ± 1	18 ± 2
PSE_568	30	60	108	6.48	0.002	6 ± 1	12 ± 2
PSE_PA01	30	60	108	5.59	0.001	6 ± 1	17 ± 3
PSE_6749	30	60	108	6.55	0.009	7 ± 1	13 ± 2
PSE_1054	30	60	108	6.01	0.002	9 ± 1	15 ± 2
PSE_1586	30	60	108	6.07	0.002	13 ± 2	22 ± 2
EKIN_502	15	60	54	6.79	0.006	1 ± 0.5	4 ± 3
EC_073	30	60	108	4.71	0.006	56 ± 4	64 ± 7
EC_042	30	60	108	1.55	0.006	74 ± 8	85 ± 9
MDR_A	140	60	504	6.88	0.002	124 ± 18	159 ± 25
MDR_B	140	60	504	6.61	0.007	185 ± 16	219 ± 22
MRSA_508^*b*^	30	60	108	6.17	0.002	12 ± 1	21 ± 3
MRSA_520^*b*^	15	60	54	6.82	0.002	1 ± 0.5	5 ± 1
MRSA_531^*b*^	30	60	108	6.41	0.001	7 ± 1	15 ± 2
MSSA_10788^*c*^	80	60	288	7.07	0.001	99 ± 12	118 ± 15
MSSA_ F77^*b*^	30	60	108	6.76	0.006	3 ± 1	12 ± 2
EFM_513	180	60	648	1.86	0.007	277 ± 16	393 ± 20
Additional S. aureus isolates (for pigmentation investigation)							
ATCC 29213^*b*^	30	60	108	6.76	0.002	5 ± 1	15 ± 2
NCTC 10442^*b*^	30	60	108	6.69	0.002	8 ± 1	20 ± 2
ATCC 33807^*c*^	80	60	288	7.01	0.002	15 ± 2	40 ± 5
NCTC 4163^*c*^	80	60	288	6.07	0.003	38 ± 5	71 ± 6

a Values are means ± standard deviations.

b Yellow pigmentation.

c Orange pigmentation.

**FIG 2:**
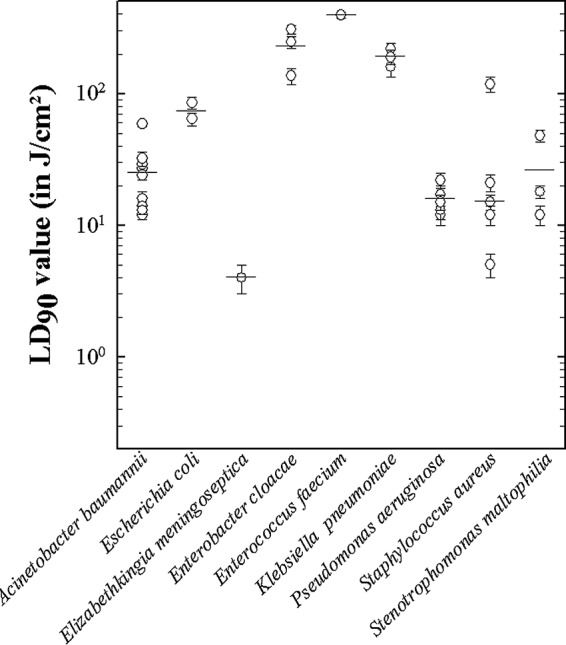
Comparison of blue light LD_90_ values between strains and species. Each individual circle represents the average LD_90_ for each strain ± the standard deviations (*n =* 3). The average LD_90_ values for the species are shown by horizontal lines.

Loss of bacterial viability associated with blue light was calculated as previously described to give LD_90_ values in terms of joules per square centimeter. Investigation of these LD_90_ values indicated that differences between the values were very likely driven by differences in the blue light susceptibilities of different bacterial species (*P* < 0.001). We found that the highest LD_90_ values belonged to E. cloacae and K. pneumoniae strains, which had values statistically higher than all other species included in the analysis (where more than one representative strain was tested) (*P* < 0.05 in all cases) (Fig. 2). The exception to this was E. coli, which had moderate blue light tolerance, but no statistical differences were seen between the strains tested. A. baumannii, S. aureus, P. aeruginosa, and S. maltophilia all had similar and low levels of resistance to the blue light exposure.

**FIG 3:**
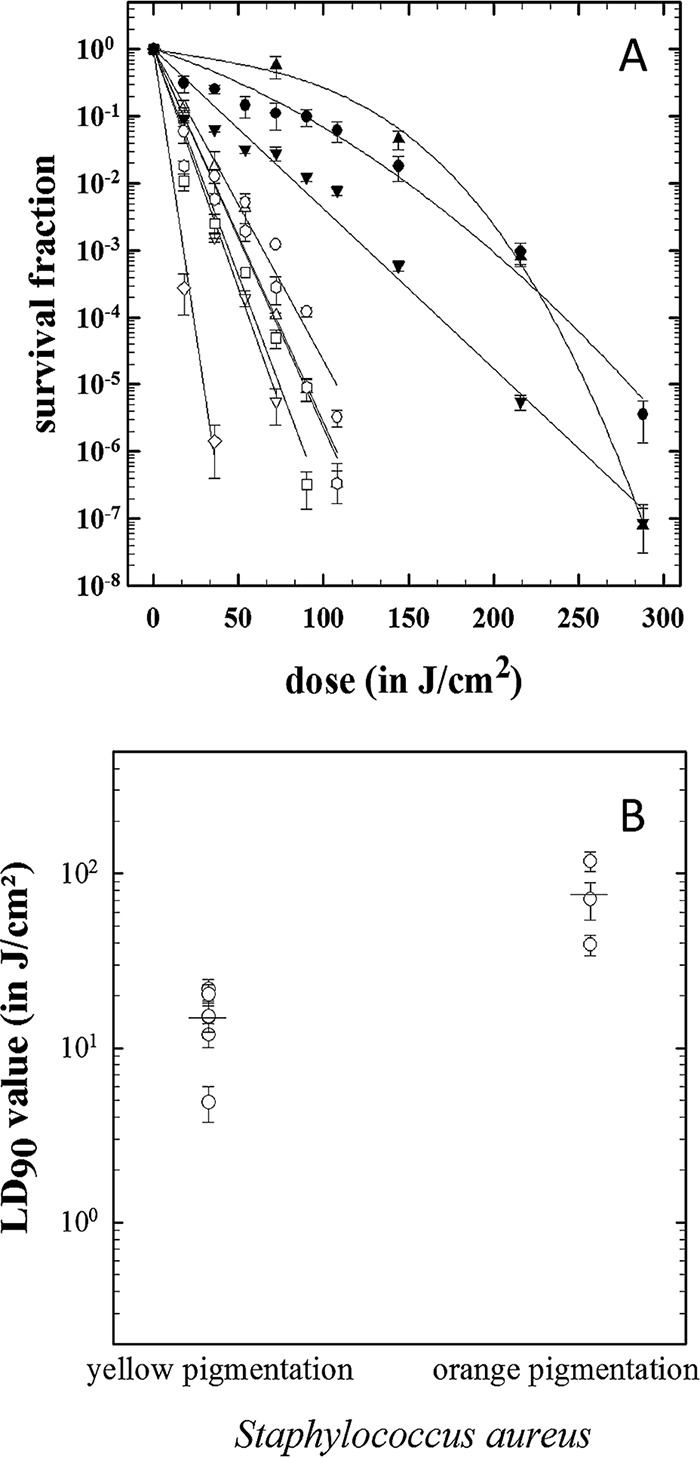
(A) Correlation between survival of planktonic S. aureus strains following blue light exposure and cell pigmentation. Orange carotenoid-producing strains are represented as follows: MSSA_4163, closed circles; MSSA_33807, closed inverted triangles; and MSSA_10788, closed triangles. Yellow non-carotenoid-producing strains are represented as follows: MSSA_10442, open circles; MSSA_F77, open squares; MRSA_520, open diamonds; MRSA_531, open inverted triangles; MRSA_508, open triangles; and MSSA_29213, open hexagons. Data are averages ± standard deviations (*n =* 3). (B) Comparison of blue light LD_90_ values between yellow- and orange-pigmented S. aureus strains. Each individual circle represents the average LD_90_ for each strain ± the standard deviation (*n =* 3). The average LD_90_ values for yellow- and orange-pigmented strains are shown by horizontal lines.

In the initial assay of planktonic cell resistance to blue light, we observed that the S. aureus strains demonstrated different colony pigmentations, appearing as either pale yellow (MRSA_508, MRSA_520, MRSA_531, and MSSA_F77) or orange (MSSA_10788) when grown on LB agar. We hypothesized that this pigmentation may be responsible for the variability seen in both the survival fraction curves and LD_90_ values when exposed to blue light (Fig. 1B and 2). Four additional culture collection strains of S. aureus were assessed for blue light sensitivity, including two pale yellow (MSSA_29213 and MSSA_10442) and two orange (MSSA_33807 and MSSA_4163) strains. In total, nine strains of S. aureus were tested, six yellow and three orange. We determined that the orange pigmentation correlated with increased resistance to blue light in both the survival fraction curves and in LD_90_ values (Fig. 3). The LD_90_ values were statistically significantly higher in the orange-pigmented strains than in their yellow counterparts (*P* = 0.003).

### Sensitivities of isolates to blue light when grown in biofilms.

**FIG 4:**
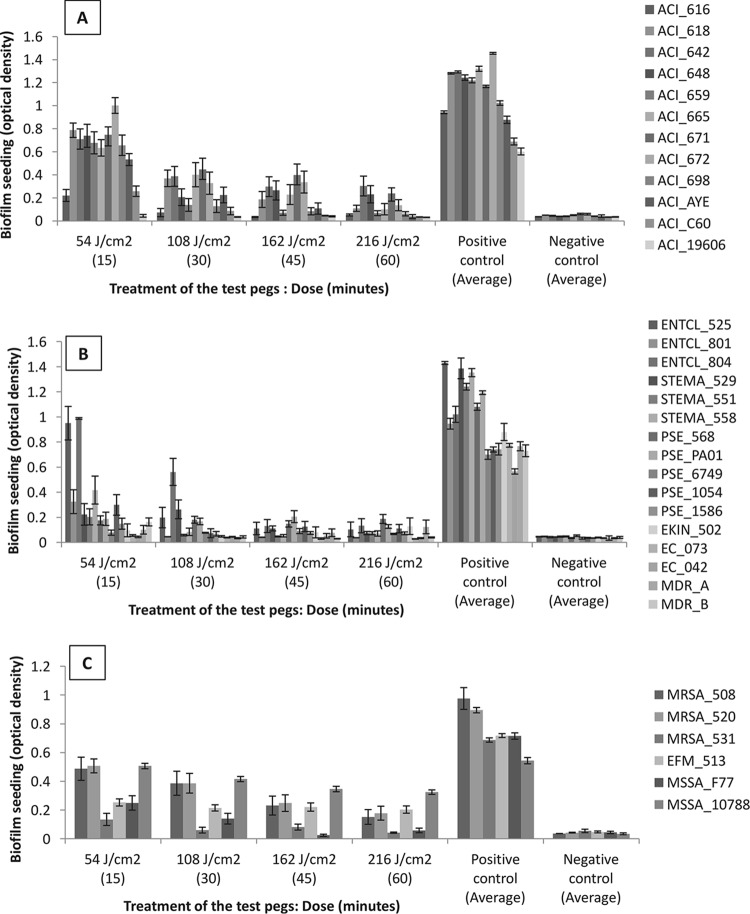
Graphs showing the biofilm seeding results for all isolates. Optical density on the *y* axis refers to the average biofilm seeding for the isolates tested after exposure to blue light at the range of durations tested (in minutes) on the *x* axis. The positive control was to the average biofilm seeding of the dark-incubated, non-blue-light-exposed isolates. The negative control was to a negative (broth-only) control. The error bars represent the standard errors.

Blue light treatment resulted in reductions in biofilm seeding for all isolates tested (Fig. 4), and the majority of these reductions (apart from the time point of 15 min for MSSA_10788) were statistically significant (*P* < 0.05 in Student's *t* tests compared to the positive control). The percentage reductions are shown in Table 3, with the single nonsignificant result indicated by a caret.

**TABLE 3:**
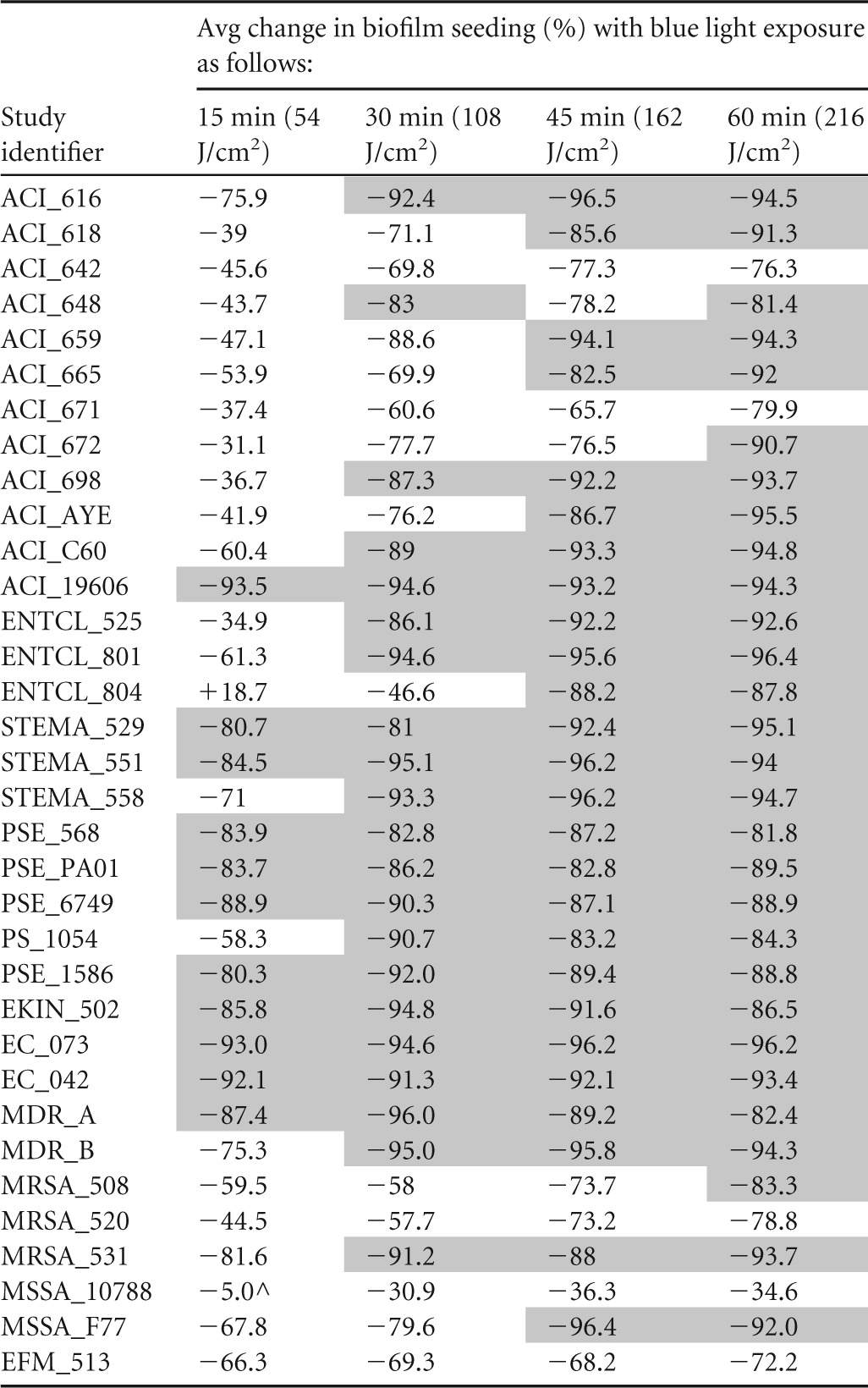
Average percent change in biofilm seeding in isolates exposed to blue light compared to nonexposed dark-incubated controls^*a*^

a Shading denotes reductions of at least 80% in biofilm seeding compared to the positive control. ^, *P* value = 0.15.

The most susceptible isolates were the Gram-negative organisms, in particular ACI_19606, for which there was a 93.5% reduction in biofilm seeding (*P* < 0.001) after 15 min (54 J/cm^2^) of blue light exposure. As a group, the other Gram-negative comparator organisms were the most susceptible, with 10/16 (63%) showing greater than 80% reductions in biofilm seeding (average = 86%) at 15 min, compared to 1/12 for A. baumannii and 1/6 for the Gram-positive organisms. Although ENTCL_804 responded well to blue light treatment for 30 min at 108 J/cm^2^ (46.6% reduction), 45 min at 162 J/cm^2^ (88.2% reduction), and 60 min at 216 J/cm^2^ (87.8% reduction), the treatment actually resulted in increased biofilm seeding at 15 min of 18.7%. This result was repeatable and was seen in a number of replicates.

As mentioned above, the Gram-positive biofilms were less susceptible to blue light treatment, with only two isolates (33%) achieving at least 90% reductions in seeding. It is important to note, however, the small sample size. One isolate of S. aureus (MSSA_10788), which is recognized in the EN standards for assessing the efficacy of chemical disinfectants, was the least sensitive to blue light, achieving a maximum reduction in biofilm seeding of 36% at 45 min (162 J/cm^2^). This result was again repeatable and was seen in 48 replicate pegs. This is further evidence toward the hypothesis that bacterial pigmentation attenuates the sensitivity to blue light in biofilms as well as in planktonic cells.

In order to characterize how the different biofilm forming properties (seeding ability and biofilm size) of each bacterial species relate to each other, a series of correlations were performed. We found no evidence for significant correlations existing between (i) median biofilm size (CV assay) and median sensitivity of biofilm to blue light (*P* = 0.133) or (ii) median biofilm size and LD_90_ (*P* = 0.912). For these reasons, we feel that any differences between species in biofilm resistance to blue light are likely to be intrinsic differences rather than a function of the biofilm. However, we are not able to dismiss the alternative hypothesis that correlations do exist, as these analyses were insufficiently powered. We found the statistical power to be 21% when considering the potential correlation between planktonic and biofilm killing (*R* = 0.200) and 33% when considering the correlation between biofilm killing and biofilm formation (*R* = −0.263). In this respect, we must actually conclude that real correlations between these parameters might exist; however, if they do exist, they are likely to less apparent than the correlation observed between biofilm formation and planktonic killing.

We found a significant correlation between the sensitivity of strains to blue light in the planktonic state and their ability to form biofilms (Spearman's coefficient = 0.369; *P* = 0.032). This indicated that strains that demonstrated greater resistance to blue light in the planktonic state were more likely to produce thicker biofilm.

## DISCUSSION

In this study, we have shown blue light (400 nm) to be effective at inactivating both planktonic cells and biofilms of important nosocomial wound pathogens. Contrary to published research (1, 15, 16), we found Gram-negative organisms to be more susceptible to blue light. There are a number of differences between these published studies and our study which may contribute to these conflicting findings. First, we tested a number of isolates per species (most of the studies test one strain of each species) comprising both clinical and control strains (most of the published studies use control strains which may have been passaged many times), and our light box exposed the bacteria to higher doses (60 mW/cm^2^) than the 10 mW/cm^2^ used by Maclean et al. (15). Although we only tested a small number of isolates, the Gram-positive biofilms appeared less sensitive to blue light treatment, with one strain (MSSA_10788) consistently resisting the effects of blue light.

Analyzing blue light susceptibility against multiple clinical strains from the same species has permitted us to assess the heterogeneity of intraspecies kill rates. In some species, such as A. baumannii, the rate of blue light-mediated killing was extremely homogeneous; however, S. aureus strains display a much more heterogeneous response to blue light stress.

It has long been recognized that bacterial cells have utilized pigmentation as a virulence factor (38). Among the most easily recognizable bacterial pigments are the triterpenoid carotenoids, which impart the eponymous golden color to S. aureus strains. Various authors have identified a correlation between strains containing the carotenoid pigment staphyloxanthin and the ability to survive on surfaces exposed to natural sunlight (39), and it is well known that carotenoids function as antioxidants. Furthermore, staphyloxanthin has been shown to provide protection for pigmented S. aureus strains against ROS produced by phagocytes (40, 41).

Augmentation of the clinical isolates of S. aureus with a series of well-characterized strains from culture collections allowed us to correlate increased blue light killing times (as seen with MSSA_10788) with colony pigmentation. Planktonic testing and assessment of the LD_90_ values per color group showed that the light sensitivity of the strongly orange-pigmented strains is significantly different from that of the standard pale yellow strains (*P* value < 0.003) (Fig. 3B). Therefore, although all species of S. aureus tested were susceptible to blue light, it is important to consider the effects of bacterial pigmentation when determining the required blue light exposure for effective decontamination.

As well as differences in sensitivity, there were also several instances where blue light treatment increased the planktonic growth and biofilm seeding. For example, with ENTCL_804, there was an increase of 18.7% in seeding after 15 min of blue light treatment. Light has been shown to facilitate growth when the wavelengths and dose are not appropriate (42), and Nussbaum et al. (43) found that wavelengths of 810 nm and 905 nm improved the growth of E. coli and S. aureus, respectively. Furthermore, Mussi et al. (44) reported that blue light treatment decreased motility and biofilm formation in A. baumannii and increased pathogenesis when cocultured with Candida albicans (a model for apoptosis in human alveolar macrophages) (45). However, there are several important differences between the studies; the fluence was considerably lower than in this study (1.32 to 1.89 mW/cm^2^ versus 60 mW/cm^2^) and the light wavelength peaked at 460 nm versus 400 nm, while the exposure was measured over 4 days versus 30 min. However, it does raise interesting questions on the effects of suboptimal light exposure on bacterial cells, which should be looked into in future studies. Furthermore, the enhanced growth in our study warrants further investigation.

We found that a correlation existed between the strains which were sensitive to blue light in the planktonic state and those which produced larger amounts of biofilm in the CV assay. The reason for this observation is not clear; however, we hypothesize that this indicates that blue light selective pressure may exist in environmental niches where protection as a biofilm might provide benefits against other stimuli that are likely to coexist with blue light. The fact that we were unable to observe a correlation between biofilm and planktonic resistance to blue light might indicate that these mechanisms are functionally independent. Planktonic cells need to rely on their intrinsic transparency, pigmentation, and repair to protect against blue light. In biofilm, bacteria can rely on more extracellular exudate and neighbors to protect against blue light.

To the best of our knowledge, our work is one of the first to show the antibacterial activity of 400-nm light against a range of clinically relevant bacterial strains (as well as control strains) and one of just a handful to look at nondental biofilms. The inclusion of multiple isolates is an additional strength of our study, as it allows correlations between phenotypic characteristics and blue light resistance to be explored. Although there are several limitations (monomicrobial biofilms tested instead of polymicrobial, the lack of formal assessment of the potential for the development for resistance, the relatively small number of isolates tested, and the potential biasing effect of the included resistant isolates), our work nonetheless provides valuable insights into this technology and especially how it relates to the eradication of biofilms for environmental decontamination.

The findings in this paper demonstrate that high-intensity blue light can be used to inactivate a wide range of clinical pathogens, not only in the planktonic state but also as mature biofilms. This technology has many practical applications within health care settings, as blue light may ameliorate opportunistic infections indirectly by reducing the bacterial load on environmental surfaces and directly within wounds. Future studies are warranted to investigate this further, especially whether the exposure times of the 400-nm blue light can be reduced for a range of different clinical applications. As blue light is equally efficacious against antibiotic-resistant pathogens, this technology may prove an important weapon in the future fight against antimicrobial resistance.

## Supplementary Material

Supplemental material
