# Supplementary material for: Antibacterial Activity of Blue Light against Nosocomial Wound Pathogens Growing Planktonically and as Mature Biofilms
Source: Appl Environ Microbiol. 2016 Jun 13;82(13):4006–16. doi: 10.1128/AEM.00756-16 (PMC4907187; doi:10.1128/AEM.00756-16)
Supplement: Supplemental material [file supp_82_13_4006__index.html]

Supplemental material 

# Antibacterial Activity of Blue Light against Nosocomial Wound Pathogens Growing Planktonically and as Mature Biofilms

## Supplemental material

- Supplemental file 1 -

  Emission spectrum of a Henkel Loctite blue-light array (Fig. S1).

  PDF, 169K
