# Supplementary material for: Antibacterial Activity of Blue Light against Nosocomial Wound Pathogens Growing Planktonically and as Mature Biofilms
Source: Appl Environ Microbiol. 2016 Jun 13;82(13):4006–16. doi: 10.1128/AEM.00756-16 (PMC4907187; doi:10.1128/AEM.00756-16)
Supplement: Supplemental material [file AEM.00756-16_zam999117232so1.pdf]

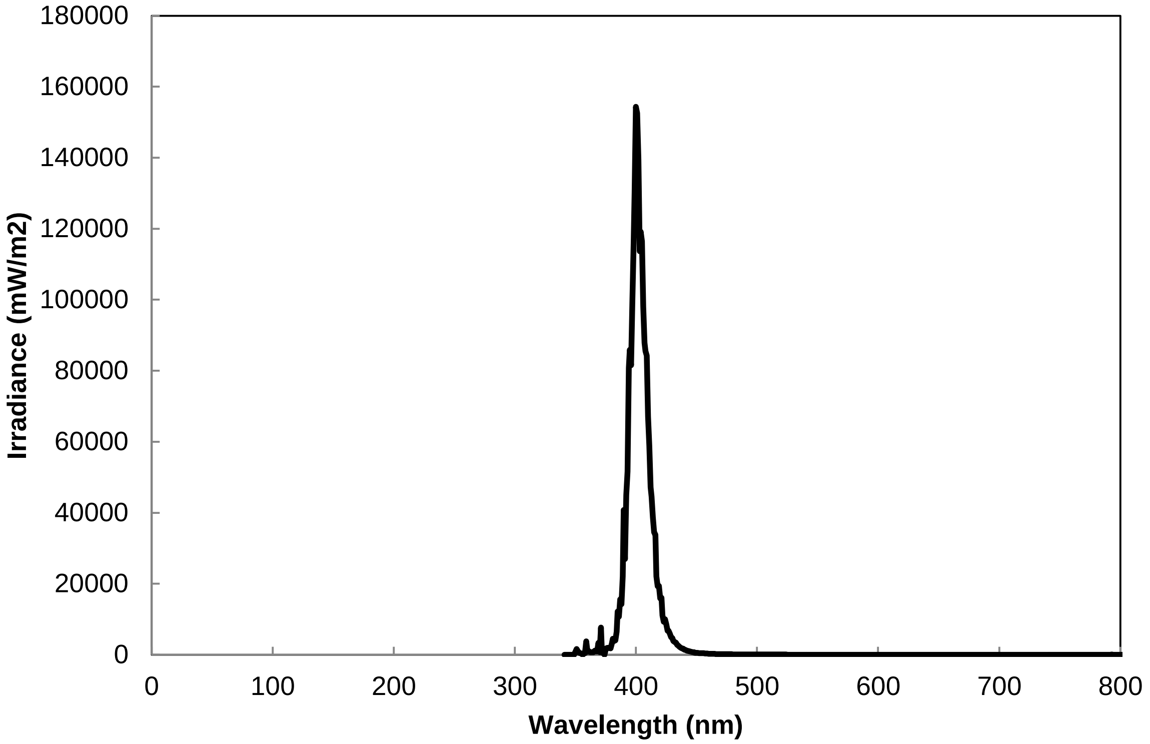

Figure S1. Emission spectrum of Henkel Loctite blue light array determined using an Ocean Optics USB2000 spectrometer
